# Molecular serotype-specific identification of *Streptococcus pneumoniae* using loop-mediated isothermal amplification

**DOI:** 10.1038/s41598-019-56225-0

**Published:** 2019-12-27

**Authors:** Chika Takano, Yoko Kuramochi, Mitsuko Seki, Dong Wook Kim, Daisuke Omagari, Mari Sasano, Bin Chang, Makoto Ohnishi, Eun Jin Kim, Kazumasa Fuwa, Paul E. Kilgore, Tomonori Hoshino, Satoshi Hayakawa

**Affiliations:** 1 Division of Microbiology, Department of Pathology and Microbiology, Nihon University School of Medicine, Tokyo, 173-8610 Japan; 2 Department of Pediatrics and Child Health, Nihon University School of Medicine, Tokyo, 173-8610 Japan; 3 Nihon University School of Medicine, Tokyo, 173-8610 Japan; 4 Department of Pediatric Dentistry, School of Dentistry, Meikai University, Saitama, Japan; 5 Department of Pharmacy, College of Pharmacy, Hanyang University, Ansan, 15588 Republic of Korea; 6 Institute of Pharmacological Research, Hanyang University, Ansan, 15588 Republic of Korea; 7 Nihon University School of Dentistry, Tokyo, 101-8310 Japan; 8 Department of Neurological Surgery, Nihon University School of Medicine, Tokyo, 173-8610 Japan; 9 Bacteriology I, National Institute of Infectious Diseases, Tokyo, 162-8640 Japan; 10 Department of Pharmacy Practice, Eugene Applebaum College of Pharmacy & Health Sciences, Wayne State University, Detroit, MI 48201 USA

**Keywords:** Bacterial infection, Meningitis

## Abstract

In children, the incidence of pneumococcal meningitis has decreased since the introduction of pneumococcal conjugate vaccine (PCV7 and PCV13). However, since the introduction of the vaccine, developed countries have seen the emergence of non-PCV13 serotypes. However, invasive pneumococcal disease (IPD) caused by PCV13-targeted serotypes still represents an important public health problem in resource-limited countries. To develop a rapid, simple, and cost-effective assay to detect serotypes of *Streptococcus pneumoniae*, we developed a novel loop-mediated isothermal amplification (LAMP) assay based on the sequences available for the 13 capsular types that are included in PCV13: 1, 3, 4, 5, 6 A, 6B, 7 F, 9 V, 14, 18 C, 19 A, 19 F, and 23 F. We evaluated test reactivity, specificity, sensitivity and performance, and compared the results between established LAMP and conventional PCR assays. To support its clinical use, the detection limits of the LAMP assay were evaluated using bacterial genomic DNA-spiked cerebrospinal fluid (CSF) and blood specimens. We confirmed the specificity of the LAMP assay using 41 serotypes of pneumococcal strains. The sensitivity of the LAMP assay was 10 to 100 copies per reaction, compared to 10 to 10^4^ copies per reaction for PCR assays. The detection limits of the LAMP assay were comparable when using DNA-spiked CSF and blood specimens, as compared to using purified DNA as the template. In conclusion, a rapid and simple LAMP-based pneumococcal serotyping method has been developed. This is the first report of a LAMP method for a PCV13 serotype-specific identification assay, which could be a promising step to facilitate epidemiological studies of pneumococcal serotyping.

## Introduction

*Streptococcus pneumoniae* is one of the primary bacterial species responsible for meningitis, bacteraemia, septicaemia, community-acquired pneumonia, and otitis media^1^. Approximately 500,000 children under the age of 5 years die of pneumococcal disease each year, with the majority of these deaths occurring in developing countries^2^. Elderly persons and immunocompromised individuals, including those with HIV/AIDS, sickle cell anaemia, cancer, and end-stage liver or kidney disease, carry the majority of the pneumococcal disease burden in developed countries. Although traditional antimicrobial therapy is effective, pneumococcal resistance to essential antimicrobials, such as penicillin, cephalosporins, and macrolides, is a serious and growing problem worldwide^3^.

Since the introduction of pneumococcal conjugate vaccine (PCV7 and PCV13), the incidence of meningitis caused by *S. pneumoniae* in children has decreased^4,5^. In developed countries, non-PCV13 serotypes have become an emerging problem since the introduction of the vaccine. However, invasive infections caused by PCV13-targeted serotypes are still a public health problem in resource-limited countries^5^. The disease burden in adults and mortality in adults and children remain high in many countries^6^. In developing countries, the adult disease burden and adult/childhood mortality rates are still high despite international collaboration, such as the Global Alliance for Vaccines and Immunization (GAVI).

Pneumococcus has highly diverse polysaccharide capsule types, containing around 94 different serotypes, and the distribution of serotypes varies geographically^7,8^. The available PCVs were designed to provide immunity against the most prevalent invasive serotypes worldwide^8^. It is crucial to understand the geographical distribution of serotypes and shifts in prevalence over time to optimize vaccine design and assess the impact of vaccine introduction on disease burden.

Conventional bacterial culture methods require a well-equipped laboratory with appropriate biosafety facilities^9^, specialized bacterial culture media, and reagents. Serological typing of pneumococci is performed using the Quellung reaction with type-specific pneumococcal antisera. In this method, serum is added to the bacteria obtained from the medium, and swelling of the capsule is scored under a microscope. Sometimes, the reaction is subtle, and its scoring requires a high degree of technical skill. Well-equipped laboratory facilities and strict control of antisera are also required. Thus, accurately determining pneumococcal serotypes remains challenging due to the limited availability of routine microbiology laboratory services and facilities in developing countries^10^.

End users in developed and developing countries require effective serotype identification tests that meet criteria to ensure global access. Cost-effective, sensitive, and specific diagnostic tests for pneumococcal serotyping are not readily available in many resource-limited countries.

In comparison to loop-mediated isothermal amplification (LAMP), multiplex polymerase chain reaction (PCR)-based assays are relatively expensive and complex to perform in resource-limited laboratory settings because they require a thermal cycler and electrophoretic analysis^11–14^. LAMP is a nucleic acid amplification method that provides rapid, accurate, and cost-effective diagnosis of infectious diseases^15,16^. LAMP-based diagnostic assays for *S. pneumoniae*, *Haemophilus influenzae*, and *Neisseria meningitidis* in cerebrospinal fluid (CSF) specimens have been established^17–20^. LAMP-based methods for meningococcal typing to detect meningococcal serogroups A, B, C, X, Y, and W^21^, as well as *H. influenzae* serotypes a, b, c, d, e, and f, have been developed^17,22^.

LAMP methods to serotype pneumococcus are not yet available. To develop a rapid, simple, and cost-effective method to detect serotypes of *S. pneumoniae*, we designed LAMP primer sets based on the sequences available for the capsular types 1, 3, 4, 5, 6 A, 6B, 7 F, 9 V, 14, 18 C, 19 A, 19 F, and 23 F (PCV7 or PCV13 vaccine-targeted serotypes).

## Results

### Analytical reactivity and specificity of LAMP-based pneumococcal serotyping

The analytical reactivity and specificity of LAMP-based pneumococcal serotyping (Table 1) were evaluated using 55 pneumococcal strains belonging to 41 pneumococcal serotypes (Table 2). A genomic DNA concentration of 10^5^ copies per reaction was used as a standard for each strain. The LAMP primer sets for capsular types 1, 3, 4, 5, 6B, 7 F, 9 V, 14, 18 C, 19 A, 19 F, and 23 F successfully amplified the target DNA sequence of each target locus (Table 1). LAMP primer sets for capsular types 1, 3, 4, 5, and 14, which are single-serotype serogroups, did not have any DNA-amplified product from DNA of other target capsular types.

**Table 1:** LAMP primer sequences in this study.

Serotyping primer name	LAMP Primer Sequence (Sequence 5′-3′; Reaction temperature, 63 °C)	Length (base pairs)	Gene/GenBank no./target serotypes
4_F3	CAT TCA GAA GTA CAA AAT TAT CAG GA	26	*wzy* /CR931635/4
4_B3	ACG CTT TAT AAC TCG GGA C	19	
4_FIP	GCT CTA ACT GCT AGT ACT GTT TTA GAT TGT ACA ATG CGG GTA GG	44	
4_BIP	TTC AGC ATA TTC AGA GGC AGC CAA GGA GAA CAC ACC AGG	39	
4_LF	ATT ACC CTA GAA ATA AAG CCC ACT C	25	
4_LB	TCA GGA ATA ATA GAT GAT TTA GGA GT	26	
6B_F3	GGG ATT GAA TTA CCG AAC AT	20	*wci*P /CR931639/6A, 6B, 6C, 6D
6B_B3	GTC CAT GTC TTC GAT ACA AGA	21	
6B_FIP	GGA ACC ATC TCT AGC AAT GCA TGA TTA GTA TTT TAT TCA TGC CTA TAT CTG G	52	
6B_BIP	CTG TCT CAT GAT A**A**^**a**^T TAT TTT GCA AAG AGT TGC TCA GGG CAG AAC	45	
6B_LF	AAA CCT GCA GTA CAC CC	17	
6B_LB	TTT GCA CTA GAG TAT GGG AAG	21	
9V_F3	AGC GAT TCG TAT TTT TGA AGA	21	*wzy* /CR931648/9V, 9A
9V_B3	TCA ACA TTG TCA GTA GCG T	19	
9V_FIP	CGG AGT TAA CGA TAA TCC CAT TTG TAA TTT TTG GTT TGG AAA AGG AC	47	
9V_BIP	TAC TAG ATA TAC TTG CTC GAA CGG GTT CCA AGA AAT AGA CTT AGA AGA AC	50	
9V_LF	CCA AGC ATT GAA ATC AAT A	19	
9V_LB	CGG GTA TTT TAT TTG TAG TG	20	
14_F3	GAG GAA TCC CTA AAA GCT AT	20	*wzy* /CR931662/14
14_B3	CAA AAT ACT GAC AAA GCT AGA	21	
14_FIP	GTG ACC CCA ATA A**A**^b^A TAT CTA CTG TAG GGA ATG GAA ATG TTA CTT GGC G	49	
14_BIP	AGG ACA GGA GTT TTA GGA AGT ATA ATA AGT CTC TCA GAT GAA TCA CA	47	
14_LF	AGG GAA TTC TGA CAC CTG	18	
14_LB	CAG TAA TGT TTT ATT ATC TG	20	
18C_F3	ATT CGA TGG CTA GAA CAG AT	20	*wzy* /CR931673/18 (18A, 18B, 18C, 18F)
18C_B3	AGC ATT TCT ATA AAG AAG AGT GT	23	
18C_FIP	TGT TAC AAA CCC TAT CCC TCT CCA AGG GAG TTG AAT CAA CCT A	43	
18C_BIP	ATG GTC TTA CAG GGA CAA TGG GTC CTA CAA ATC CTA TCT CAA TGT	45	
18C_LF	CCA TAA ATA TAG GGG CGA	18	
18C_LB	GAT CCA TAA TGA TAT TTT GAA GTA C	25	
19F_F3	TGG ATT TGT TGG TTT AAT AGC AG	23	*wzy* /CR931678/19F
19F_B3	GAT AAT TAA CTA GGC CCA TTT CC	23	
19F_FIP	CAC TCT CAA ATA GCG TCC TAG TCG GTA TTC CAG CAT TTT ACT ACT CTT	48	
19F_BIP	GAG GCT CAA TTC AGC ATT TTA ATC AGG CAC CAA TGT TTC ACT G	43	
19F_LF	TGA ACG ACC GGC TAA AAA CA	20	
19F_LB	AGA TCC TGG TGA AGT TTT TGG	21	
23F_F3	GGC GTT AAC ATT TTT TTT CAA AC	23	*wzy* /CR931685/23F
23F_B3	CAA CTA ACC CAA CAT AAC CAT	21	
23F_FIP	GCA TCC CCA AAA AAC AAA TGA AAC CAA TCA TAT AGC CAT CGA GTG	45	
23F_BIP	CCT TTG GAA ATA CGA CGA AGG GGT AAA GGC ATC TCT ACC GTT	42	
23F_LF	AAA AAA ATT CAC AAC ACC T	19	
23F_LB	TGG ACA CAA TAT TAG AAG TG	20	
1_F3	CAG CTA GTC GTA ATT TAC AGA T	22	*wzy* /CR931632/1
1_B3	TTA CAA TTC CAA AGT ATC CTC C	22	
1_FIP	CCA AGT TTG ATT AGA ATA CCC CGT GCA ATT ATT TCG AAG GTC GT	44	
1_BIP	TGG ACA CCT TTT TTC CAA ACG TTT CAC ATA TCC CTC TCC CAC	42	
1_LF	CTT ACT ATG TCA TTA AAA AAA G	22	
1_LB	TCA ACC AAA TAT GGT TTT ACT CT	23	
3_F3	GCC TGT TAG ATA TGA AGA TGT TTC	24	*gal*U/CR931634/3
3_B3	ATG TAT CAA TAG CAT CTG TCA AT	23	
3_FIP	TTG GTT TCT CTA CAA AAG CAT CAA CTT CTT ATG GTG TGA TTT CTC CT	47	
3_BIP	TTG GAC GTT ATC TAC TTA CTC CTG ATT CAT TAC CTG CTC CTG G	43	
3_LF	AGA GGC CAT TAC TAC TTT CCA ATC	24	
3_LB	GAT TTT TTC TAT ATT AGA AAC CCA	24	
5_F3	CCC ATG ATT TAT GCC CTC T	19	*wzy* /CR931637/5
5_B3	TGT TTC AGA ATG TTC ACC AAC	21	
5_FIP	GGC ATT GAC AGT ATA AGA AAA AGC ACA ACG TTC TTC TTC TCA TCG T	46	
5_BIP	TTG AAG GTT ACG CGC CAT TTG TGT ATT CAG AAG GCA ACC	39	
5_LF	GGG CTA AAA AAA GCA TGC GGA	21	
5_LB	GGT GCC AAG AGT TTT ATT CTT TGG	24	
7F_F3	ATT ATT TGG CTA TTC AAC AGG A	22	*wzy* /CR931643/7F, 7A
7F_B3	GAA CAA TCC TAT AAA TCC ATT CTC A	25	
7F_FIP	AGT CTG CCA AAC ATC TCC ATA AAA CTA GTT CTG ATT TTG GTC GG	44	
7F_BIP	AGA GGC GGA AAT TTC AAA AAT TCC GTG AAC AGA TAG TAA TGG GTG TA	47	
7F_LF	GAG ATT ATT TGA ACA ATT GAA CT	23	
7F_LB	GAT ATT TAG TGG TTC C	16	
19A_F3	AGC TCT TAC TAT TAT AGT TGA CCT	24	*wzy* /CR931675/19A
19A_B3	GAG CGT TTA TGA CTA TAA ATG AAG A	25	
19A_FIP	GAA CCA CTG AAA ATT TGA ACC CGT TAG GAG AGA GAT TCA TAA TCT TGC	48	
19A_BIP	TAC CAG TTA TGA AGG TGA GCT AAC ATC CAA AAA TAT AAG CAG ATA CGT	48	
19A_LB	GTG CGA ACT TCG ATT CGG G	19	

^a^point mutation related to serotype switching.

^b^original sequence was T (to avoid formation of primer dimer and nonspecific reactions).

**Table 2:** Reactivity and specificity of the pneumococcal serotyping LAMP assay.

Vaccine	Serotypes	No. of strains	Strain ID	Origin of isolate	LAMP primer set											
					4	6B	9V	14	18C	19F	23F	1	3	5	7F	19A
PCV7	4	2	SP0852, SP0143	RT^a^, CSF	+^b^	—	—	—	—	—	—	—	—	—	—	—
	6B	2	SP0857, SP1489	N, N	—	+	—	—	—	—	—	—	—	—	—	—
	9V	2	SP0916, SP2928	N, B	—	—	+	—	—	—	—	—	—	—	—	—
	14	2	SP0869, SP3320	N, B	—	—	—	+	—	—	—	—	—	—	—	—
	18C	2	SP0873, SP2818	N, B	—	—	—	—	+	—	—	—	—	—	—	—
	19F	2	SP0862, SP1118	N, N	—	—	—	—	—	+	—	—	—	—	—	—
	23F	2	SP0885, SP2838	N, N	—	—	—	—	—	—	+	—	—	—	—	—
PCV13	1	2	SP3121, SP3070	B, B	—	—	—	—	—	—	—	+	—	—	—	—
	3	2	SP1441, SP3256	N, N	—	—	—	—	—	—	—	—	+	—	—	—
	5	2	SP3034, ATCC6305	U, U	—	—	—	—	—	—	—	—	—	+	—	—
	6A	2	SP1567, SP1589	N, N	—	+	—	—	—	—	—	—	—	—	—	—
	7F	2	SP3365, SP3172	B, RT	—	—	—	—	—	—	—	—	—	—	+	—
	19A	2	SP1516, SP3081	N, N	—	—	—	—	—	—	—	—	—	—	—	+
PPSV23	2	1	D39	U	—	—	—	—	—	—	—	—	—	—	—	—
	8	1	ATCC6308	U	—	—	—	—	—	—	—	—	—	—	—	—
	9N	1	SP2700	CSF	—	—	—	—	—	—	—	—	—	—	—	—
	10A	1	SP1933	N	—	—	—	—	—	—	—	—	—	—	—	—
	11A/E	1	SP2760	RT	—	—	—	—	—	—	—	—	—	—	—	—
	12F	1	SP0113	B	—	—	—	—	—	—	—	—	—	—	—	—
	15B	1	SP3354	N	—	—	—	—	—	—	—	—	—	—	—	—
	17F	1	NCTC11904	U	—	—	—	—	—	—	—	—	—	—	—	—
	20	1	SP2830	N	—	—	—	—	—	—	—	—	—	—	—	—
	22F	1	SP1854	N	—	—	—	—	—	—	—	—	—	—	—	—
	33F	1	SP3201	N	—	—	—	—	—	—	—	—	—	—	—	—
Non- vaccine serotypes	6C	1	SP3362	N	—	+	—	—	—	—	—	—	—	—	—	—
	6D	1	SP2739	N	—	+	—	—	—	—	—	—	—	—	—	—
	7A	1	2040/37	U	—	—	—	—	—	—	—	—	—	—	+	—
	7C	1	SP3285	N	—	—	—	—	—	—	—	—	—	—	—	—
	9A	1	Wilder	U	—	—	+	—	—	—	—	—	—	—	—	—
	13	1	SP0073	N	—	—	—	—	—	—	—	—	—	—	—	—
	15A	1	SP2758	RT	—	—	—	—	—	—	—	—	—	—	—	—
	15C	1	SP3343	N	—	—	—	—	—	—	—	—	—	—	—	—
	18A	2	SP0852, 8609/43	B, U	—	—	—	—	+	—	—	—	—	—	—	—
	18B	1	SP1901	N	—	—	—	—	+	—	—	—	—	—	—	—
	18F	1	Lederle	U	—	—	—	—	+	—	—	—	—	—	—	—
	23A	1	SP3374	N	—	—	—	—	—	—	—	—	—	—	—	—
	24F	1	SP3193	N	—	—	—	—	—	—	—	—	—	—	—	—
	34	1	SP3359	N	—	—	—	—	—	—	—	—	—	—	—	—
	35B	1	SP3357	N	—	—	—	—	—	—	—	—	—	—	—	—
	37	1	SP2742	N	—	—	—	—	—	—	—	—	—	—	—	—
	38	1	SP3356	N	—	—	—	—	—	—	—	—	—	—	—	—

^a^RT, respiratory tract specimen; CSF, cerebrospinal fluid; N, nasopharyngeal swab; B, blood; U, unknown.

^b^+, Amplification within 25 min incubation; −, no amplification within 120 min incubation.

The LAMP primer set for capsular type 6B also amplified DNA of capsular types 6 A, 6 C, and 6D. DNA of other capsular types was not amplified. Capsular types 6 A, 6B, 6 C, and 6D share almost the same sequence of putative rhamnosyl transferase gene (*wciP*)^23^. An important point mutation in this sequence is related to serotype switching, e.g. capsular type 6B changes to 6 A^24^. To detect capsular types 6 A, 6B, 6 C, and 6D, we designed the backward inner primer (BIP) corresponding to the middle of the B1 region where the important mutation is found (Table 1).

The LAMP primer set for capsular type 7 F amplified DNA of capsular type 7 A. Capsular types 7 A and 7 F share the same sequence of serotype7A/7F-specific oligosaccharide repeat unit polymerase (*wzy*)^25^. DNA of other capsular types, including capsular type 7 C, was not amplified. Bentley *et al*. reported that the *wzy* sequences of capsular types 7B and 7 C differ from those of capsular types 7 A and 7 F^25^. The polymerization linkage of *wzy* in types 7B and 7 C is D-Glcp(β1–4)D-Glcp; in types 7 A and 7 F, it is D-Glcp(β1–3)D-GalpNAc.

The LAMP primer set for capsular type 9 V amplified DNA of capsular type 9 A. Capsular types 9 A and 9 V share the same sequence of serotype 9 A/9V-specific *wzy* gene. DNA of other target capsular types, including the capsular type 9 N, was not amplified. Capsular type 9 N has a different *wzy* sequence^25^. The polymerization linkage of *wzy* in types 9 A and 9 V is D-Glcp(β1–4)D-Glcp, and D-Glcp(β1–4)D-GlcpNAc in type 9 N^25^.

The LAMP primer set for capsular type 18 C amplified DNA of capsular types 18 A, 18B, and 18 F. Capsular types 18 A, 18B, 18 C, and 18 F share the same sequence of the serotype 18 A/18B/18 C/18F-specific *wzy* gene^25^. DNA of other capsular types was not amplified.

The LAMP primer set for capsular type 19 A targeting the *wzy* gene for serotype 19 A detected capsular type 19 A only. DNA of other capsular types, including 19 F, was not detected by this primer set. Likewise, the LAMP primer set for capsular type 19 F that targeted the *wzy* gene for serotype 19 F detected capsular type 19 F only. DNA of other target capsular types, including 19 A, was not detected by this primer set. The *wzy* genes for 19 A and 19 F have different sequences^25^.

Pimenta *et al*. reported difficulty in differentiating between capsular types 19 A and 19 F when they used PCR-based pneumococcal serotyping methods^13^. We designed a LAMP primer set corresponding to the specific region of each capsular type after alignment analysis of *wzy* sequences for the two capsular types. The LAMP primer set consisted of six primers including eight regions of the target sequences, while the PCR primer sets consisted of only two primers (forward and reverse primers) including two regions of the target sequences. Therefore, in comparison to the PCR primers, the LAMP primer set should easily distinguish between those two capsular types.

The LAMP primer set targeting the *wzy* gene for capsular type 23 F detected capsular type 23 F only. DNA of other capsular types, including capsular type 23 A, was not detected, as the *wzy* genes for 23 A and 23 F have different sequences^25^.

LAMP-amplified products were analysed by direct DNA sequencing to confirm the specificity. The obtained sequences were compared with those of the target region of the original sequence at each capsulation locus (between F1 and B1; Supplementary Fig. S1), and identity to the expected nucleotide sequences was confirmed (Fig. S2).

### Detection limit of LAMP-based pneumococcal serotyping method

**Figure 1:**
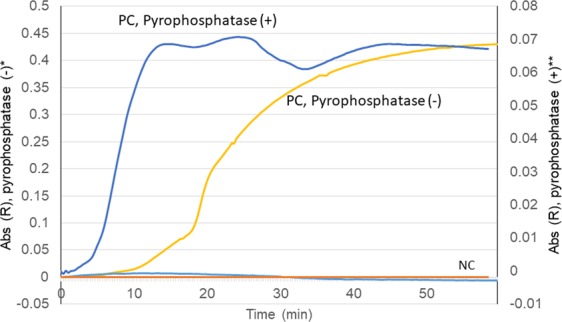
The relationship between reaction time and the absorbance of the reaction tubes. Colorimetric dye can be used with pyrophosphatase. Pyrophosphate, a by-product of the LAMP reaction, attenuates the activity of DNA polymerase. The addition of pyrophosphatase can increase the LAMP reaction speed. *, Bst DNA Polymerase (New England Biolabs, Ipswich, MS, USA) & conventional LAMP reagent; **, Isothermal Master Mix (no dye; Canon Medical Systems Corporation, Tochigi, Japan); PC, positive control; NC, negative control.

The detection limits of the LAMP assay were 10 genome copies per reaction for capsular types 14, 18 C, 19 F, and 23 F; and 10^2^ genome copies per reaction for capsular types 1, 3, 4, 5, 6B, 7 F, 9 V, and 19 A. The detection limits of the PCR assay were 10 genome copies per reaction for capsular type 19 F; 10^2^ genome copies per reaction for capsular types 4, 6B, 7 F, 9 V, 14, 18 C, 19 A, and 23 F; 10^3^ genome copies per reaction for capsular types 1 and 3; and 10^4^ genome copies per reaction for capsular type 5 (Table 3). For six of the serotypes, the sensitivity of the LAMP assay was 10- to 100-fold greater than that of PCR-based pneumococcal serotyping. The products were visually inspected by monitoring the turbidity/colour of the reaction tube and utilizing real-time turbidimetry and a real-time colorimetric sensor (Fig. 1). The detection limits of each serotyping LAMP assay were identical in the real-time measurement and direct visual inspection. No LAMP amplification was detected in control samples lacking target DNA. The experiments were repeated in triplicate over 3 days, and identical results were obtained in laboratories both in Japan and South Korea.

**Table 3:** Detection limits of LAMP and PCR assays detecting DNAs of pneumococcal serotypes and using the DNA spiked CSF specimens.

*S.pneumoniae* serotypes	Detection limit (Purified DNA)		Detection limit (DNA spiked CSF^a^)		Detection limit (DNA spiked blood)	
	PCR^b,c^	LAMP^b^	PCR	LAMP	PCR	LAMP
4	10^2^copies ^d^	10^2^	10^2^	10^2^	>10^5^	10^2^
6B	10^2^	10^2^	10^3^	10^2^	10^5^	10^2^
9V	10^2^	10^2^	10^2^	10^2^	>10^5^	10^2^
14	10^2^	10	10^3^	10	10^5^	10
18C	10^2^	10	10^2^	10	>10^5^	10^2^
19F	10	10	10	10	10^3^	10
23F	10^2^	10	10^3^	10	>10^5^	10^2^
1	10^3^	10^2^	10^3^	10^2^	>10^5^	10^2^
3	10^3^	10^2^	10^3^	10^2^	10^5^	10^2^
5	10^4^	10^2^	10^4^	10^2^	>10^5^	10^2^
7F	10^2^	10^2^	10^2^	10^2^	>10^5^	10^2^
19A	10^2^	10^2^	10^2^	10^2^	>10^5^	10^2^

^a^Cerebrospinal fluid specimen collected between 1998 and 2002^39^.

^b^PCR results were obtained by electrophoretic analysis. LAMP results were determined visually.

^c^conventional PCR (serotypes 1, 3, 4, 5, 6B, 18C, 19F and 23F^11^; serotypes 9V, 7F^12^; serotype 14^14^; serotype 19A^13^).

^d^Number of genome copies per reaction.

### LAMP analysis of DNA-spiked specimens

The LAMP assay detected 10–100 genome copies of the genomic DNA even when using DNA-spiked CSF specimens as the template. The results were identical to those obtained with purified DNA as the template (Table 3). The detection limits of PCR-based pneumococcal serotyping assay for serotypes 6B, 14, and 23 F decreased from 10^2^ to 10^3^ genome copies per reaction. The detection limits for other serotypes were the same as when purified DNA was used as the template.

Using DNA-spiked blood specimens, the detection limits of the LAMP assay were slightly attenuated from 10 copies to 100 copies for serotypes 18 C and 23 F; other serotypes were detected with sensitivity as high as with purified DNA as the template. In contrast, the detection limits of all PCR-based pneumococcal serotyping assay changed from 10–10^3^ to 10^3^–10^5^ or more genome copies per reaction (Table 3). Again, the results of the LAMP assay were identical between direct visual inspection and real-time measurement.

### LAMP assays using colorimetric visual inspection dye plus a real-time colorimetric sensor

The results of LAMP assay using colorimetric visual inspection dye and a real-time colorimetric sensor are shown in Fig. 1. The colour change of the LAMP reagents was readily observed. Detection time of the LAMP reaction decreased from 19 minutes to 8 minutes when the colorimetric dye was used with pyrophosphatase (Isothermal Master Mix, no dye; Canon Medical Systems Corporation, Tochigi, Japan). This reaction protocol can increase the LAMP reaction speed.

## Discussion

In children under 5 years old, the incidence of non-vaccine serotype-specific invasive pneumococcal diseases has increased worldwide because of the widespread introduction of PCV7 or PCV13^5,26^. In adults, the number of clinical infections with such non-vaccine pneumococcal-serotype-specific strains has also increased^6^. In Japan, PCV usage has reduced antibiotic resistance^27^, while the most common serotypes of pneumococcal strains are still PCV7 or PCV13 vaccine-targeted strains. At the same time, diseases due to *S. pneumoniae* that were not included in the vaccines (such as serotypes 8, 10 A, 12 F, 15 A, 23 A, and 24 F) have been reported^5,28^.

To optimize vaccine design and assess the impact on disease burden following vaccine introduction, it is crucial to understand the geographic distribution of serotypes and shifts in prevalence over time. Although such studies are typically conducted in central laboratories with equipment and qualified personnel, the future application of nucleic acid detection methods such as LAMP have potentially important roles in diagnosing a number of prevalent pathogens, particularly in low-resource countries. *S. pneumoniae* is one of these pathogens. The use of standard pneumococcal serotyping assays is limited in developing countries, and the accurate determination of pneumococcal serotyping remains a challenge^10^. For these reasons, future studies are anticipated to compare serotyping classification and accuracy using traditional methods with LAMP methods. The Quellung reaction using type-specific pneumococcal antisera and a microscope, the conventional serological typing of pneumococcus, requires microbiology laboratory services, expensive antisera and experienced technician.

Varying degrees of success have been achieved using multiplex PCR-based assays for pneumococcal serotyping. However, in contrast to LAMP assays, PCR requires thermal cyclers, electrophoresis, UV lamps and technicians with expertise in using equipment to run PCR assays.

The pneumococcal serotype-specific LAMP assay showed good performance in differentiating each target pneumococcal capsular type. The pneumococcal serotype-specific LAMP assay was analytically specific and had a better detection limit compared to conventional pneumococcal serotype-specific PCR. The high sensitivity of this pneumococcal serotype-specific LAMP assay is consistent with previous studies^17–19,21^.

To conduct a pilot evaluation of the pneumococcal serotyping LAMP assay, we used two methods for DNA preparation. CSF specimens were simply heated and centrifuged. A commercially available kit (Procedure for Ultra Rapid Extraction, PURE; Eiken Chemical) was used for the blood. PURE can produce a DNA solution suitable for the LAMP reaction within 10 minutes without the use of a centrifuge. It can be used with dried blood spots, which can be extended to field study^29^.

The pneumococcal serotype-specific LAMP reaction demonstrated equivalent sensitivity with spiked CSF samples and purified DNA template. LAMP reactions were not inhibited, or were inhibited only slightly, when using DNA-spiked CSF and blood. PCR is inhibited by biological substances, particularly heparin^30^ and other blood components, including haem, leukocyte DNA, and immunoglobulin G^22,31,32^. The LAMP assay can be performed using simple DNA preparation methods because the LAMP reaction more readily tolerates potentially disturbing biological elements (i.e. reaction inhibitors) than PCR^33^.

The LAMP method requires only the preparation of a reaction mixture and placement of the tube into some incubator at 63 °C. Amplicon of the target DNA can be detected easily by visual inspection, with no requirement for specialized equipment to read the results. Furthermore, due to its high sensitivity and robustness of the reaction, DNA sample preparation can be simplified, such as by boiling or using the PURE method.

The robustness, superior detection limit and simple performance of the LAMP assay make it an excellent alternative to pneumococcal serotype-specific PCR^34^. Although the detection accuracy should be further improved, the convenience of the LAMP assay could facilitate surveillance of pneumococcal serotypes compared to PCR.

This study assessed pneumococcal serotype-specific LAMP products using a reaction mixture including pyrophosphatase. In the reaction, rather than a “white precipitate”, we observed a colour change from colourless to violet due to triphenylmethane dye, which binds to double-stranded DNA (D-QUICK; Kaneka Co., Osaka, Japan), indicating a positive reaction^35^. To determine the LAMP amplification results, we usually observe a white precipitate of magnesium pyrophosphate, which is the by-product of LAMP reaction. As an alternative, we observed a colour change of the reaction mixture using a thermostatic colour sensor (MyAbscope^®^; Kaneka Co., Osaka, Japan) that measures the absorbance of the reaction mixture in real time. As shown in Fig. 1, the detection time was reduced when pyrophosphatase was used in the reaction mixture, compared to using conventional LAMP reagents without pyrophosphatase. Pyrophosphate, a by-product of the LAMP reaction, attenuates the activity of DNA polymerase, and addition of pyrophosphatase can increase the LAMP reaction speed.

This is the first report of a PCV13 pneumococcal serotype-specific identification assay using the LAMP method. Using this method, decreases in PCV13 serotypes associated with vaccine use can easily be observed. Development of the serotype-specific LAMP assay represents a promising step to facilitate epidemiological studies of pneumococcal serotyping. Based on this study, further LAMP-based methods are currently under preparation for PPSV23 targets and other serotypes predicted to emerge after the widespread use of PCV13. This study represents the first step toward achieving our ultimate objective of developing LAMP-based methods covering all pneumococcal serotypes.

## Materials and Methods

### Bacterial strains

In this study, we analysed 55 strains of *S. pneumoniae*, including serotypes that belong to PCV7, PCV13, and PPSV23, and a number of non-vaccine serotypes. The PCV7 serotypes were serotype 4 (SP0852, SP0143), serotype 6B (SP0857, SP1489), serotype 9 V (SP0916, SP2928), serotype 14 (SP0869, SP3320), serotype 18 C (SP0873, SP2818), serotype 19 F (SP0862, SP1118), and serotype 23 F (SP0885, SP2838). Additional PCV13 serotypes were serotype 1 (SP3121, SP3070), serotype 3 (SP1441, SP3256), serotype 5 (SP3034, ATCC6305), serotype 6 A (SP1567, SP1589), serotype 7 F (SP3365, SP3172), and serotype 19 A (SP1516, SP3081). PPSV23 serotypes were serotype 2 (D39), serotype 8 (ATCC6308), serotype 9 N (SP2700), serotype 10 A (SP1933), serotype 11 A/E (SP2760), serotype 12 F (SP0113), serotype 15B (SP3354), serotype 17 F (NCTC11904), serotype 20 (SP2830), serotype 22 F (SP1854), and serotype 33 F (SP3201). Non-vaccine serotypes were serotype 6 C (SP3362), serotype 6D (SP2739), serotype 7 A (2040/37), serotype 7 C (SP3285), serotype 9 A (Wilder), serotype 13 (SP0073), serotype 15 A (SP2758), serotype 15 C (SP3343), serotype 18 A (SP0852, 8609/43), serotype 18B (SP1901), serotype 18 F (Lederle), serotype 23 A (SP3374), serotype 24 F (SP3193), serotype 34 (SP3359), serotype 35B (SP3357), serotype 37 (SP2742), and serotype 38 (SP3356) strains. By Quellung reaction using type-specific pneumococcal antisera (Statens Serum Institute, Copenhagen, Denmark), the capsule production of the 54 reference strains was identified in advance. Strain SP2760 was indicated as serotype 11 A/E because serotypes 11 A and 11E could not be discriminated by the Quellung reaction.

### Preparation of chromosomal DNA

Chromosomal DNA from the 55 strains was prepared using a Wizard^®^ Genomic DNA Purification Kit (Promega, Fitchburg, WI, USA) according to the manufacturer’s recommendations. The concentration of chromosomal DNA was measured with a NanoDrop 1000 spectrophotometer (Thermo Fisher Scientific Inc., Waltham, MA, USA). The genome copy number was estimated based on the molecular size of *S. pneumoniae* strain R6 (2.0 Mbp; GenBank accession number, NC_003098). Each DNA sample was diluted to 10^5^ DNA copies/reaction and used to evaluate the specificity of assays. For the detection limit study, serial tenfold dilutions of genomic DNA from PCV13 serotypes (capsular types 1, 3, 4, 5, 6B, 7 F, 9 V, 14, 18 C, 19 A, 19 F, and 23 F, which are SP0852, SP0857, SP0916, SP0869, SP0862, SP0885, SP3121, SP1441, SP3034, SP1567, SP3365, and SP1516, respectively) were amplified by LAMP, and we then compared the results with those of conventional PCR^11–14^. To determine the detection limit, triplicate LAMP testing of *S. pneumoniae* was carried out using serial tenfold dilutions of chromosomal DNA over a 3-day period. The supernatant of pooled *S. pneumoniae*-negative CSF specimens was used in the spiking assay^36^. Tenfold dilutions of each serotype of *S. pneumoniae* genomic DNA were amplified using the established LAMP and conventional PCR assays^11–14^.

### LAMP primer design

As shown in Table 1, 12 LAMP primer sets for *S. pneumoniae* were designed with reference to the published sequences in GenBank and using LAMP primer design software^37^. The LAMP primer set for *S. pneumoniae* consisted of two outer primers (F3 and B3), a forward inner primer (FIP), a backward inner primer (BIP), and loop primers (LF and/or LB).

### LAMP reaction

The LAMP procedure used in this study was described previously^22^. Briefly, we carried out LAMP in a reaction mixture consisting of 1.6 µM each of FIP and BIP, 0.2 µM each of F3 and B3, 0.4 µM of LF/LB, 8 U of *Bst* DNA polymerase large fragment (New England Biolabs, Ipswich, MA, USA), 1.4 mM deoxynucleoside triphosphates, 0.8 M betaine (Sigma, St. Louis, MO, USA), 20 mM Tris-HCl (pH 8.8), 10 mM KCl, 10 mM (NH_4_)_2_SO_4_, 8 mM MgSO_4_, 0.1% Tween 20, and template DNA, and the final volume was adjusted to 25 µL with distilled water. We incubated each reaction mixture at 63 °C for 60 minutes and then heated at 80 °C for 2 minutes for termination of the reaction.

### Analysis of LAMP products

The turbidity of the reaction tube was determined in real-time by reading the optical density at 650 nm (OD_650_) at 6-s intervals using a Loopamp^®^ real-time turbidimeter (LA-500; Eiken Chemical Co., Tokyo, Japan). We calculated the amplification time required to exceed a turbidity of 0.1 (*Tt*) using the turbidimeter software, as described previously^38^. The detection limit was measured using a colorimetric visual inspection dye (leucotriphenylmethane^35^; D-QUICK, Kaneka Co., Osaka, Japan), Isothermal Master Mix (no dye; Canon Medical Systems Corporation, Tochigi, Japan), and a thermostatic colour sensor (MyAbscope^®^; Kaneka Co., Osaka, Japan). The colour change in the reaction mixture was examined at intervals of 20 s. Amplification time was determined with the thermostatic colour sensor software, as when the OD_650_ exceeded 0.01.

Each amplified LAMP product was sequenced at Akita Prefectural University Biotechnology Centre using a BigDye^®^ Terminator V3.1 cycle sequencing kit (Applied Biosystems, Foster City, CA, USA) and a 3130xL Genetic Analyser (Applied Biosystems), and their sequences were verified using the primers shown in the Supplementary Table.

### PCR assay

PCR was carried out in 25-µL reaction mixtures containing 1 U Ex Taq DNA polymerase (TaKaRa Bio, Tokyo, Japan), 0.2 mM of each deoxyribonucleoside triphosphate, 10 mM Tris-HCl buffer (pH 8.3), 50 mM KCl, 2 mM MgCl_2_, 0.5 µM of each primer, and 2 µL of template DNA. Amplification was carried out with two thermal cyclers: Veriti™ (Applied Biosystems, Foster City, CA, USA) and T-100™ (Bio-Rad, Hercules, CA, USA) for 35 cycles of denaturation at 95 °C for 30 s, primer annealing at 54 °C for 90 s, and extension at 72 °C for 60 s, with a final incubation at 72 °C for 10 minutes^11–14^. The PCR products were electrophoresed on agarose gels and visualized by staining with ethidium bromide.

### DNA-spiked clinical CSF and blood specimens

As a pilot evaluation of the pneumococcal serotyping LAMP assays, 39 pneumococcal PCR-negative specimens were randomly chosen from CSF collected in a previous bacterial meningitis study in Hanoi, Vietnam^36^. The specimens of CSF were incubated at 95 °C for 2 minutes as pre-treatment and centrifuged at 13,000 × *g* for 5 minutes. The supernatants of these specimens were kept for use in DNA-spiked CSF experiments.

Blood was collected from five healthy volunteers. Blood samples were heparinized for storage. Using Procedure for Ultra Rapid Extraction (PURE; Eiken Chemical, Tokyo), DNA from the blood samples was prepared and used for DNA-spiked blood experiments.

### Ethical declaration

We used CSF specimens preserved from our previous surveillance study^36,39^. All CSF specimens used in this study were de-identified prior to laboratory processing and analysis. Ethical approval for patient specimen collection during surveillance was obtained from the following ethics review committees: The Institutional Review Board (IRB) of the International Vaccine Institute, Seoul, South Korea, and the IRB at the National Institute of Hygiene and Epidemiology, Hanoi, Vietnam. Each institution participated in prospective, population-based surveillance of childhood meningitis from 1999 to 2002^36,39^. During these surveillance studies, written consent was not obtained as CSF collection was considered routine standard care for hospitalized children with suspected bacterial meningitis. Therefore, verbal consent from the parent or legal guardian present with the child during the period of hospitalization was recorded in the patient’s medical chart at the time of the clinical lumbar puncture procedure. This consent procedure was approved by the local scientific ethics review committees of the participating institutions.

We received informed consent prior to collecting blood from five healthy volunteers. The procedures were approved by the IRB of Nihon University School of Medicine (IRB No. 28-9-0/1). All experiments were performed in accordance with relevant guidelines and regulations.

## Supplementary information

Supplementary information 
