# Supplementary material for: Molecular serotype-specific identification of Streptococcus pneumoniae using loop-mediated isothermal amplification
Source: Sci Rep. 2019 Dec 27;9:19823. doi: 10.1038/s41598-019-56225-0 (PMC6934563; doi:10.1038/s41598-019-56225-0)
Supplement: Supplementary file 1 — Supplementary information [file 41598_2019_56225_MOESM1_ESM.pdf]

Supplementary Materials

Molecular serotype-specific identification of *Streptococcus pneumoniae* using loop-mediated isothermal amplification

Chika Takano[1, 2]*, Yoko Kuramochi[3]*, Mitsuko Seki[1, 4], Dong Wook Kim[5, 6], Daisuke Omagari[7], Mari Sasano[1, 8], Bin Chang[9], Makoto Ohnishi[9], Eun Jin Kim[5, 6], Kazumasa Fuwa[2], Paul E. Kilgore[10], Tomonori Hoshino[4] & Satoshi Hayakawa[1]

[1]Division of Microbiology, Department of Pathology and Microbiology, Nihon University School of Medicine, Tokyo, 173-8610, Japan; [2]Department of Pediatrics and Child Health, Nihon University School of Medicine, Tokyo, 173-8610, Japan; [3]Nihon University School of Medicine, Tokyo, 173-8610, Japan; [4]Department of Pediatric Dentistry, School of Dentistry, Meikai University, Saitama, Japan; [5]Department of Pharmacy, College of Pharmacy, Hanyang University, Ansan, 15588, Republic of Korea; [6]Institute of Pharmacological Research, Hanyang University, Ansan, 15588, Republic of Korea; [7]Nihon University School of Dentistry, Tokyo, 101-8310, Japan; [8]Department of Neurological Surgery, Nihon University School of Medicine, Tokyo, 173-8610, Japan; [9]Bacteriology I, National Institute of Infectious Diseases, Tokyo, 162-8640, Japan; [10]Department of Pharmacy Practice, Eugene Applebaum College of Pharmacy & Health Sciences, Wayne State University, Detroit, MI, 48201, USA.

*These authors contributed equally to this work.

Correspondence and requests for materials should be addressed to M.S. (email: mitsuko.seki@dent.meikai-u.ac.jp) and D.W.K. (email: dongwook@hanyang.ac.kr)

Fig. S1. Nucleotide sequence of the *S. pneumoniae* serotype-specific genes used to design the pneumococcal serotyping LAMP primers. The sequences used for the LAMP primers are indicated by arrows. (a) to (l) in the figure are the sequences for *S. pneumoniae* serotype-specific genes for serotypes 4, 6B, 9V, 14, 18C, 19F, 23F, 1, 3, 5, 7F, and 19A, respectively.

Fig. S1 (a) Target, serotype 4; GeneBank no. CR931635; Gene, *wzy*

```
No.      9677                                                                    9746
sequence TTGCTTTCTA ATTATTTTTC AAATTACTGG TTTTATTTTA CAAAAAGTTA GTATATATGA TTTTTCTGTA

No.      9747                                                                    9816
sequence TGGTATCTGA TTTTATCTTA TTTTTTTATG TTTGGATTAA TTTTCAATGA GTATATGGGG TTTCAAACAA

No.      9817                                                                    9886
sequence CTCTGCTGTG GAGCCCTAGT AACTTCTATA ATAATGAAGA ATTATTTCAT TCATATATTT TTATAATTTG

No.      9887                                                                    9956
sequence GATTTTGTTT TGTTATTCTG TAGGCTATTT ATTTTTTTAT AGTGATGGAA AGGTACATTA TCATTCAGAA
primer                                                                      |--------

No.      9957                                                                   10026
sequence GTACAAAATT ATCAGGAAAA TGAAGAGAAA ATTTTGTACA ATGCGGGTAG GATTTTAACA GGAGTGGGCT
primer   -------F3-------->                  |--------F2-------->           <--------

No.      10027                                                                  10096
sequence TTATTTCTAG GGTAATAACT GATTCTAAAA CAGTACTAGC AGTTAGAGCG GCGAATAGCT ATTCAGCATA
primer   -------LF-------|        <------------F1------------|              |--------

No.      10097                                                                  10166
sequence TTCAGAGGCA GCTAGTTCAG GAATAATAGA TGATTTAGGA GTACTTATGC TTCCTGGTGT GTTCTCCTTG
primer   -----B1----->    |------------LB------------->           <--------B2-------|

No.      10167                                                                  10236
sequence TTTTATTCAG ATAAGCTGTC CCGAGTTATA AAGCGTACTA TTTTTTGGGT AATGTTGTTT TACCTAATTT
primer                     <--------B3---------|

No.      10237                                                                  10306
sequence TGATAATGAT CCTAACAGGA AGTCGTAAAA TCCAGGTGTT TTCAATCTTG GCGTTGGTTT TAGTATACAC

No.      10307                                                                  10376
sequence ACAATCTTTA GGGATTACTT TTTCGAAGAA AAGAGTACTT GTATTTTTAA TAGTAACTGT ATTTCTATTA
```

Fig. S1 (b) Target, serotype 6A, 6B, 6C, 6D; GeneBank no. CR931639; Gene, *wciP*

```
No.        8410                                                                   8479
sequence   GGAGCTTATT ACAATTTTTT TAATCTAATT GAATACGTTA AAAACAATTA TCAATTTGAT TATTACTTTT

No.        8480                                                                   8479
sequence   TTTGTGATCA AGATGATATT TGGAAAGAGC ACAAGTTAGA AATACAGCTG TTAAGATTTT CTAAAGATGA

No.        8550                                                                   8619
sequence   CATGCCAGAG ATGGTTTACT CTGATATGTC AACGATTGAT GCCAGTAATA ATTTGATAGA TATTAGTATA

No.        8620                                                                   8689
sequence   AATAAAATAA TGGGGATTGA ATTACCGAAC ATAAATAATT TGTATTTTAT TCATGCCTAT ATCTGGGGGT
primer                  |---------F3--------->          |-----------F2------------><---

No.        8690                                                                   8759
sequence   GTACTGCAGG TTTTAATCAT GCATTGCTAG AGATGGTTCC TTCAGTTGAT ATTGATAAAG ATTATTTATA
primer     ------LF-----|<-----------F1--------------|

No.        8760                                                                   8829
sequence   TATAGAAAAA CTGTCTCATG ATAATTATTT TGCAAAGTTT GCACTAGAGT ATGGGAAGGT GTTGTTCTGC
primer                |------------B1-------------->|---------LB---------->      <------

No.        8830                                                                   8899
sequence   CCTGAGCAAC TGGTCTTGTA TCGAAGACAT GGACATAATG TAACAACTAG TCATCATTTT AAATTATCTC
primer     ----B2-----|  <---------B3----------|

No.        8900                                                                   8969
sequence   CGCTAAATGT TTTCAGAAAG GCTATATTGG GTTTCAATGA ATTGGCACTT ACACATGCTG GGGTATATAA

No.        8970                                                                   9039
sequence   TCAAACTCTT TATATGCTAA AAAAAGCTTC TGGAAAAAAT CCTTTAAGTG ATAGACTACT TGAAATTCAG
```

Fig. S1 (c) Target, serotype 9V, 9A; GeneBank no. CR931648; Gene, *wzy*

```
No.         10306                                                                  10375
sequence    GTTCGGAATT TTATCATTTT TAACACAATC CAGTACAGCG ATTATATGTT ATGTGCTTTT TATTTTTATG

No.         10376                                                                  10445
sequence    CGTATGTTAG GTTTAAAGGA AAATATAGGA AAACCATATT CTTTAGTCAG TATAACTGTG TATATTTGTT

No.         10446                                                                  10515
sequence    TTAGTTTTTC TATAATTTTT TCACAAAGTA CAATTTTATC AACTTTTACA GCAATTTTTT CCAAAAATGC

No.         10516                                                                  10585
sequence    AACCTTTTCA GGACGTATCA ATATTTGGCA GTTAGCGATT CGTATTTTTG AAGAAAATTT TTGGTTTGGA
primer                                          |---------F3----------> |------F2-------

No.         10586                                                                  10655
sequence    AAAGGACTTA ATATTGATTT CAATGCTTGG ACAAATGGGA TTATCGTTAA CTCCGCACAC AATACCTTAC
primer      ------>     <-------LF---------| <-----------F1------------|             |--

No.         10656                                                                  10725
sequence    TAGATATACT TGCTCGAACG GGTATATTTC CGGGTATTTT ATTTGTAGTG GTTCTTCTAA GTCTATTTCT
primer      -----------B1---------->         |--------LB---------> <---------B2---------

No.         10726                                                                  10795
sequence    TGGAAAATAT AGAGTAAAAT CAAAAACGCT ACTGACAATG TTGATTTCAT TCATGGTTTA TATTACGATG
primer      ----|                      <--------B3---------|

No.         10796                                                                  10865
sequence    GAGGCTACAT CTGTAAGTAT TCTTCTATTA ATAATTGCTA TTTGTGTGTA TTGGTCTTTT GGAGAGGAGA

No.         10866               10888
sequence    AGTTATATGA GCAAGTTACT TAA
```

Fig. S1 (d) Target, serotype 14; GeneBank no. CR931662; Gene, *wzy*

```
No.       7661                                                                      7730
sequence  ATTTAGATTT TTTACACAAA CTATCATTTT TGCTTTTCTT CTAGAGGTTT TGGTACTAAG CAAATCTAGA

No.       7731                                                                      7800
sequence  ATAGCGATTG TTGCAATGCT TATATATATA GCATTTGCAG TAGTCAATGA GATTAATTCA AACAATAAAT

No.       7801                                                                      7870
sequence  GGCTTATTGG AATTTTCTGT CCAATTATTC CTTTTATGTT ATTTTACAAT TTTGAAAAAA TTAAACAGAT

No.       7871                                                                      7940
sequence  TTTTTTTCAA ATGTTTAGTT CTAGATCGGG TAGCAATGCG ACACGCTTTA GAGTGTATGA GGAATCCCTA
primer                                                                   |-----F3-----

No.       7941                                                                      8010
sequence  AAAGCTATTA ATGGAATGGA AATGTTACTT GGCGCAGGTG TCAGAATTCC CTCTACAGTA GATATATTAT
primer    ------->     |----------F2----------><--------LF--------|<--------F1--------

No.       8011                                                                      8080
sequence  TGGGGTCACA TTCTATGTAT ATAAGTTTTA TTTATAGGAC AGGAGTTTTA GGAAGTATAA TAATAACAGT
primer    --------|                             |-------------B1-------------->   |---

No.       8081                                                                      8150
sequence  AATGTTTTAT TATCTGTTTT CTAAATTTTT AAAATGTGAT TCATCTGAGA GACTAAGAAG TATTGGCTAT
primer    ------LB-------->                    <---------B2--------|

No.       8151                                                                      8220
sequence  ATTCTAGCTT TGTCAGTATT TTGGCTTTTT GAAGAGTTAG ATCCACATTA TTGGTGTTTA ATTTTATTTT
primer      <----------B3---------|

No.       8221                                                    8273
sequence  TTTCAACAAT AAGTATTTTC ATAAACAATA GAAAAGAGGA AATAGTTGGA TGA
```

Fig. S1 (e) Target, serotype 18 (18A, 18B, 18C, 18F); GeneBank no. CR931673; Gene, *wzy*

```
No.       12718                                                                  12787
sequence  CTTAAACGAA TCGTTCTTTT GAGTGTATTA ATTATCATAC CAGTATTTTT GGTAATTTAT TGGTATGATA

No.       12788                                                                  12857
sequence  AAAAAGTAAG TAAACTAGGG AAAGAACGAA AAATTTTAAG TTTATTAAAT ATCTTTTCCT TAATATTTAT

No.       12858                                                                  12927
sequence  AACAGGAATA TTCCTTTATG TTTATAGTGT AAAATCTGAT TTTATATATA CATTTATTCA AGAACATAAT

No.       12928                                                                  12997
sequence  ATTAATTCGA TGGCTAGAAC AGATTTATGG AAGGGAGTTG AATCAACCTA TAATTTCGCC CCTATATTTA
primer        |---------F3--------->       |---------F2-------->      <-------LF------

No.       12998                                                                  13067
sequence  TGGGGAGAGG GATAGGGTTT GTAACAAAAT GGATGGATAA TAATTGGATG ACTTTGAATA TCAATGGTCT
primer    --|<----------F1-----------|                                         |------

No.       13068                                                                  13137
sequence  TACAGGGACA ATGGGGATCC ATAATGATAT TTTGAAGTAC TACATTGAGA TAGGATTTGT AGGATTATTT
primer    ------B1------->|-----------LB------------>  <----------B2-----------|

No.       13138                                                                  13207
sequence  ATTTATTTTT ACACTCTTCT TTATAGAAAT GCTAAACGTA TATTTGTAAA AATTGGTCAT AAAGAATCAT
primer               <----------B3-----------|

No.       13208                                                                  13277
sequence  TCATATATTT TGTATTGATA ATGTTTCAGA TGCTGATATG GTTTACAGAT AATATTTCAA TTTACCATAA

No.       13278                                                                  13347
sequence  TTTTTTATGG ATCCTAAATC TACTACTCTT TTCTTTAACT AATTCGGATA CAGAATTGGA AAATTTAGAT
```

Fig. S1 (f) Target, serotype 19F; GeneBank no. CR931678; Gene, *wzy*

```
No.       11370                                                                  11439
sequence  ATATTTTTGG ATATACTTTT ATTAATAATT TTCTTATCTA TCGCCATAAA CGCCCTAAAG ACATATTACT

No.       11440                                                                  11509
sequence  TTTAGTACCT TTATTAATAT TTATTTCAAA AACTTTAATA TCAGGAGGCC GGCAAGATAT TATTAAAATT

No.       11510                                                                  11579
sequence  CTGATTGCCT ATGTAATCAT GATGTATATC CAACAAAAAC GGAAAGTTGG ATGGAATAGA GTCATATCTC

No.       11580                                                                  11649
sequence  ATAAATATAT TCACCTTGGA TTTGTTGGTT TAATAGCAGG TATTCCAGCA TTTTACTACT CTTTGTTTTT
primer                     |----------F3----------->|------------F2-----------><------

No.       11650                                                                  11719
sequence  AGCCGGTCGT TCAACGACTA GGACGCTATT TGAGAGTGTT TCGACCTATC TAGGAGGCTC AATTCAGCAT
primer    -----LF------| <-----------F1-----------|                 |-------B1--------

No.       11720                                                                  11789
sequence  TTTAATCAGT ATATTGAAAA TCCATTAGAT CCTGGTGAAG TTTTTGGCAG TGAAACATTG GTGCCTATAT
primer    ------->                    |----------LB---------><-------B2---------|

No.       11790                                                                  11859
sequence  TAAATATATT AGGGGAAATG GGCCTAGTTA ATTATCGTAG TACAATTCAT TTAGAATTTC GGACACTAGG
primer                  <-----------B3----------|

No.       11860                                                                  11929
sequence  AGTTACTGTA GGAAATGTTT ATACTTTTTT TAGAAGACCC TTGCATGATT TTGGTCTAGT TGGTATGTAT

No.       11930                                                                  11999
sequence  GTATTTGTCT TTGCTGTAGG TGCTTTTTTT GCTATTTATT ATTTAGTTCT GAGAAAGAAA CAGGTTGGTT
```

Fig. S1 (g) Target, serotype 23F; GeneBank no. CR931685; Gene, *wzy*

```
No.       8841                                                                      8910
sequence  CAATTACAAT TTTAGTTTCC TATATTCTAC TATATGTCAG CAGAAAATAT GACGCTAAAC ATCAGATTGA

No.       8911                                                                      8980
sequence  TAGTTTTGTA TTATGGTTAG ATCTTTTTTT ACTTTTAATA TCTAATACGC GAACAGTTTA TATAATACTA

No.       8981                                                                      9050
sequence  GTTGTTTTTT GGATTATTAT TAATAGAAAT TTTATAAATA ATATTAAAAA AGAGCATAGA CTGGTAGTGA

No.       9051                                                                      9120
sequence  CAGCAACGAC AATAGTCATC TCTTTACTGG CGTTAACATT TTTTTTCAAA CATATAATTA ATAATAGTGA
primer                                  |-----------F3----------->                   |

No.       9121                                                                      9190
sequence  ATCATATAGC CATCGAGTGT TAGGTGTTGT GAATTTTTTT AAATATTATG AATCAGATAG GTTTCATTTG
primer    --------F2--------->   <--------LF--------|                    <------------

No.       9191                                                                      9260
sequence  TTTTTTGGGG ATGCTGAATT AGCCTTTGGA AATACGACGA AGGGTTATGG ACACAATATT AGAAGTGTGT
primer    --F1----------|         |----------B1---------->   |---------LB--------->

No.       9261                                                                      9330
sequence  TAGGTTGGGA TGGAACGGTA GAGATGCCTT TACTCAGTGT AATGATTAAA AATGGTTATG TTGGGTTAGT
primer                  <---------B2---------|                    <--------B3---------

No.       9331                                                                      9400
sequence  TGGTTACATT ATAGTGCTGT TTAAGTTTAT ATCTTCAATA ATCTCAGTTA AAAATAGCAC AAAAAAAAAT
primer    -|

No.       9401                                                                      9470
Sequence  ATTGGATTAT CAATCTTTAT TCCGTTACTT CTATCTGCAA CAGTTGAAAA TTATATCGTC AACATTAGCT

No.       9471                                                                      9540
sequence  TTGTATTCAT GCCTGTCTGC TTTTGTATTT TATGTTCAAT TAAAAATATA AAATTAGTAA ATAACAGAAA
```

Fig. S1 (h) Target, serotype 1; GeneBank no. CR931632; Gene, *wzy*

```
No.       10334                                                                  10403
sequence  ACAGGGGAAA GAGATTATAT GTTTACTTTA TTCATCCTTG ATGTGATTAT ATTATTTTAC TTTCACAAAA

No.       10404                                                                  10473
sequence  TAAAACGTTT TTATCTTGTA ATGTTAGTTC CGCTTGCGGC ACTACTGATT CCGTTATCTG CAGTTTTTAA

No.       10474                                                                  10543
sequence  ATATACTTTA TTAAGTGGGC AAGTTTCATC TATAAATACA AATAATATTT GGTTTGATTT GCTAGATGGT

No.       10544                                                                  10613
sequence  GAGTTTGTAT CAGCTAGTCG TAATTTACAG ATTCTGATAT TACATAATAT GGGCAATTAT TTCGAAGGTC
primer               |----------F3---------->                      |---------F2-------

No.       10614                                                                  10683
sequence  GTTCTTTTTT TAATGACATA GTAAGAATTT TTTATAATAC GGGGTATTCT AATCAAACTT GGTTTATGGA
primer    -> <---------LF-----------|              <-----------F1------------|    |---

No.       10684                                                                  10753
sequence  CACCTTTTTT CCAAACGTAC ATTCAACCAA ATATGGTTTT ACTCTTGTGG GAGAGGGATA TGTGAATGGA
primer    --------B1-------->     |----------LB-----------> <---------B2---------| <--

No.       10754                                                                  10823
sequence  GGATACTTTG GAATTGTAAT GATTTTTATG CTAACAGGCT TTTTGATGAG ATTTCTTTA TATAAACGCTC
primer    --------B3---------|

No.       10824                                                                  10893
sequence  AAAGAAATAT ATACGGGATG CTGATATATC TGTATATGAT ACCTATTTTT ATTTATTCTA CTCGTGCTGA

No.       10894                                                                  10963
sequence  TTTTGCTAAC ATATTATCTC CATTACTTAA ATATGCTATA TTGGGAACAT TGGTTATTGT TTTTATCAAT
```

Fig. S1 (i) Target, serotype 3; GeneBank no. CR931634; Gene, *galU*

```
No.       8783                                                                      8852
sequence  CTGATATTAA AGTACATTTC GTTCGTCAAA GTTCACCACG TGGTCTTGGT GACGCTGTTC TCCAAGCGAA

No.       8853                                                                      8922
sequence  GTCTTTTGTT GGTGACGATC CCTTTGTTGT AATGCTTGGT GATGACCTTA TGGATATCAC CGACTCAACT

No.       8923                                                                      8992
Sequence  GCTGTACCTT TAACAAGACA ATTGATGGAT GATTACAACG CAACACAGGC TTCAACTATC GCAGTAATGC
primer                                                                              |-

No.       8993                                                                      9062
sequence  CTGTTAGATA TGAAGATGTT TCTTCTTATG GTGTGATTTC TCCTAGATTG GAAAGTAGTA ATGGCCTCTA
primer    ----------F3----------->|----------F2----------> <-----------LF-----------|

No.       9063                                                                      9132
sequence  TAGTGTTGAT GCTTTTGTAG AGAAACCAAA ACCAGAAGAA GCGCCTAGCA ATTTAGCTAT TATTGGACGT
primer        <-----------F1------------|                                     |-------

No.       9133                                                                      9202
sequence  TATCTACTTA CTCCTGAGAT TTTTTCTATA TTAGAAACCC AAAAGCCAGG AGCAGGTAAT GAAATTCAAT
primer    --------B1------->|------------LB-----------> <-------B2---------|     <-

No.       9203                                                                      9272
sequence  TGACAGATGC TATTGATACA TTGAATAAGA CACAGAGTGT TTTTGCGCGT GAATTTGTGG GCAAACGTTA
primer    ---------B3-----------|

No.       9273                                                                      9342
sequence  TGATGTTGGT GATAAGTTTA ATTTTATGAA AACATCAATT GATTATGCTC TTCAACATCC TCAGATTAAA

No.       9343                                                                      9412
sequence  GAGAGTTTAA AAAATTACGT TATTGCACTT GGTAAGCAAT TGGAGAAGCT AGATGACTGT TCGTCAAGTG
```

Fig. S1 (j) Target, serotype 5; GeneBank no. CR931637; Gene, *wzy*

```
No.        6773                                                                     6842
sequence   TATCAATAAT CTATTTTTAA GGGTCACATC TGAATATCGA CCATCTAGTC TGACTTTGGA CTACGTTTCC

No.        6843                                                                     6912
sequence   TCCAACCGTT TTGTGTATTT TGAGATCTTT GCTAAAGAAA TTTCAGGACA TGAATTAACA GGGATTGGTT

No.        6913                                                                     6982
Sequence   ATTATTATAT GGATAATTTT TTTCTAGAGA GTTTTCTCAA TTATGGTTAT ATAGTAGGGA CTGCTTTTGT

No.        6983                                                                     7052
sequence   TTTAATAGCC TTGTCTCCCA TGATTTATGC CCTCTTGCAA CGTTCTTCTT CTCATCGTTT CCGCATGCTT
primer                      |----------F3------->  |----------F2---------> <------LF---

No.        7053                                                                     7122
sequence   TTTTTAGCCC TTGCTTTTTC TTATACTGTC AATGCCTTAT TTGAAGGTTA CGCGCCATTT GGTCCTGGTG
primer     ---------|  <-----------F1------------|     |---------B1-------->       |---

No.        7123                                                                     7192
sequence   CCAAGAGTTT TATTCTTTGG TTAGTTTTTG GTTGCCTTCT GAATACACGA ATTGGAAAGG TTGGTGAACA
primer     ---------LB--------->          <---------B2--------|            <-----------

No.        7193         7208
sequence   TTCTGAAACA AGTTAG
primer     ---B3----|
```

Fig. S1 (k) Target, serotype 7F, 7A; GeneBank no. CR931643; Gene, *wzy*

```
No.        15065                                                                  15134
sequence   ATTATGTATC AGATAATTCG AGTTTAGTTA TTTTAACAAG TTATATCGTT TCAACTCTAT ACGTAGCTGC

No.        15135                                                                  15204
sequence   TTTGTCAGAG TTAAAGATTA TTTTTGTGGA ATTACCTATT ATTATTATTT TGACTTTACT ATTTAAAAGA

No.        15205                                                                  15274
Sequence   TTAGGAATAA AAATACTGCT TAAAATAATA TCTATCACTT GCATAGTGGT GGTCGCTTTG GCAATCTCAA

No.        15275                                                                  15344
sequence   TTCCTATTTT ATATGAATTA TTCCCTATAT TTGACAATTT TTTTAAATTA GAAAAATTAT TTGGCTATTC
primer                                                                 |-------F3------

No.        15345                                                                  15414
sequence   AACAGGAAAC TATTCAACTA GTTCTGATTT TGGTCGGTTA AGTTCAATTG TTCAAATAAT CTCAACTATT
primer     ------>           |---------F2--------->    <----------LF-----------|

No.        15415                                                                  15484
sequence   TTTTATGGAG ATGTTTGGCA GACTTTATTT GGTATTGGTT TAGGAGAGGC GGAAATTTCA AAAATTCCGA
primer     <----------F1------------|                      |-----------B1----------->|-

No.        15485                                                                  15554
sequence   TATTTAGTGG TTCCTTTTAT TATAATTACG AATACACCCA TTACTATCTG TTCACTTTAT CATATGTTTT
Primer     -------LB----->                    <-----------B2----------|

No.        15555                                                                  15624
sequence   TATTGAGAAT GGATTTATAG GATTGTTCCT ATTCATGTTT ATGCCAATTT ACCTTGCTTT AAAGATGATT
primer        <-------------B3----------|

No.        15625                                                                  15694
sequence   TTTGAGCGAG GGACATCTAA ATATGATCTT CTTCTGATAG TATTATCTAT TTCTTTATTT ATGCTCTTGT

No.        15695                                                                  15764
sequence   TGTATAATAA TACAATGAGG TCAGAGATAT ATTATCTTTA TTTATTTGTT CTTTCATGGG GAGTTGCAAT
```

Fig. S1 (l) Target, serotype 19A; GeneBank no. CR931675; Gene, *wzy*

```
No.         9327                                                                     9396
sequence    AAGATTTGAT AGCTCCGCCA GTTGTCATGT CTGTTATGTT TCTCATTAGT TCTGTATTTG CCCTTATTAA

No.         9397                                                                     9466
sequence    TGTGCAAAAC TGGAATATTG AGTATAGTGG TTTAGCTTAT CTTTTGATTA TTAGTGGCAT TATTGTTTTT

No.         9467                                                                     9536
Sequence    TCAATGCCTC TCTTGGCTCT TAATTCGCCT AGTCTAAATA CCAAGATTAA AGTGACGGAT CGGCTCATTG

No.         9537                                                                     9606
sequence    ATATCCAATT CTGGAAAATA GCTCTTACTA TTATAGTTGA CCTCATTATT CTATATCTTT ATAGGAGAGA
primer                          |-----------F3------------>                    |--------

No.         9607                                                                     9676
sequence    GATTCATAAT CTTGCACTTA GCCATGGTTA TACGGGTTCA AATTTTCAGT GGTTCTTTAG AAATGCTACC
primer      ----F2--------->                  <------------F1----------|            |---

No.         9677                                                                     9746
sequence    AGTTATGAAG GTGAGCTAAC AGTGCGAACT TCGATTCGGG TCCTCATTCG TATCATTGAC GTATCTGCTT
primer      ----------B1---------->|--------LB-------->                    <------B2----

No.         9747                                                                     9816
sequence    ATATTTTTGG ATATACTTTT ATTAATAATT TCTTCATTTA TAGTCATAAA CGCTCTAAAG ATTTACTGCT
Primer      -----------|                     <------------B3-----------|

No.         9817                                                                     9886
sequence    CTTAGTTCCA TTCTTGATTT TTATTTCTAA AACCTTATTA TCTGGGGGTA GATTGGATAT TATAAAAATT

No.         9887                                                                     9956
sequence    TTAATTGCGT ATGTTGTAAT GGCCTATATT CAGCAAAAAC GAAAAGTTGG CTGGGATAAG GTCATCTCCC
```

Fig. S2. Sequence data of amplified products of the pneumococcal-serotype-specific LAMP. (a) to (l) in the figure are the sequences for the amplified products of *S. pneumoniae* serotype-specific genes for serotypes 4, 6B, 9V, 14, 18C, 19F, 23F, 1, 3, 5, 7F, and 19A, respectively.

(a)
TCTAAAACAG TACTAGCAGT TAGAGCGGCG AATAGCTATT
CAGCATATTC AGAGGCAGC

(b)
TAATCATGCA TTGCTAGAGA TGGTTCCTTC AGTTGATATT
GATAAAGATT ATTTATATAT AGAAAAACTG TCTCATGATA
ATTATTTTGC AAAG

(c)
CCCGTTCGAG CAAGTATATC TAGTACACAC AATACCTTAC
TAGATATACT TGCTCGAACG GG

(d)
CTACAGTAGA TATTTTATTG GGGTCACATT CTATGTATAT
AAGTTTTATT TATAGGACAG GAGTTTTAGG AAGTATAATA A

(e)
GGAGAGGGAT AGGGTTTGTA ACAAAATGGA TGGATAATAA
TTGGATGACT TTGAATATCA ATGGTCTTAC AGGGACAATG GG

(f)
CGACTAGGAC GCTATTTGAG AGTGTTTCGA CCTATCTAGG
AGGCTCAATT CAGCATTTTA ATCA

(g)
GGTTTCATTT GTTTTTTGGG GATGCTGAAT TAGCCTTTGG
AAATACGACG AAGGG

(h)
ACGGGGTATT CTAATCAAAC TTGGTTTATG GACACCTTTT
TTCCAAACGT

(i)
GTTGATGCTT TTGTAGAGAA ACCAAAACCA GAAGAAGCGC
CTAGCAATTT AGCTATTATT GGACGTTATC TACTTACTCC TGA

(j)
TGCTTTTTCT TATACTGTCA ATGCCTTATT TGAAGGTTAC
GCGCCATTT

(k)
TTTTATGGAG ATGTTTGGCA GACTTTATTT GGTATTGGTT
TAGGAGAGGC GGAAATTTCA AAAATTCC

(l)
ACGGGTTCAA ATTTTCAGTG GTTCTTTAGA AATGCTACCA
GTTATGAAGG TGAGCTAACA
